# Rapidly Progressive Ocular Proptosis as the First Sign of Neuroblastoma in a 16-Month-Old Child: Case Report and Review of Literature

**DOI:** 10.7759/cureus.20982

**Published:** 2022-01-06

**Authors:** Rahaf A Mandura

**Affiliations:** 1 Ophthalmology, King Abdul-Aziz University, Jeddah, SAU

**Keywords:** orbital metastasis, malignancy, infant, ocular proptosis, neuroblastoma

## Abstract

Neuroblastoma (NB) is a malignant neoplasm accounting for 7.5% of malignancy in children. It can originate anywhere along the sympathetic chain with the adrenal medulla being the most common site in 35% of cases. The initial presentation of orbital metastasis is very unusual that accounts for only 8% of all NB cases. We report a rare case of a 16-month-old girl who initially presented with bilateral rapidly progressive ocular proptosis for two weeks. CT scan of the brain and orbits revealed bilateral heterogeneous lateral orbital lesions, and CT scan of the abdomen and pelvis revealed huge heterogeneous right adrenal lesions. Histopathology of the abdominal mass confirmed the diagnosis of stage IV NB with orbital metastasis and the patient was started on an aggressive chemotherapy regimen. Ophthalmologists have a vital role in the diagnosis of NB which should be considered in the differential diagnosis of any rapidly progressive proptosis in young children. Early investigation and systemic workup should be made immediately, as it is a potentially life-threatening malignant tumor that requires aggressive management.

## Introduction

Neuroblastoma (NB) is a malignant tumor of early childhood that accounts for 7.5% of all cancers in children younger than 15 years of age and is responsible for 15% of tumor-related deaths in children [1]. It is considered an embryonal neuroendocrine tumor that originates from the neural crest progenitor cells [2]. Approximately 1% of patients with NB present initially with evidence of metastatic disease without a readily identifiable primary lesion [3]. The primary tumor can arise anywhere along the sympathetic chain, but the adrenal medulla is a common site (35%). Gene markers are being explored to determine the causes of having such aggressive initial presentations in young children [3]. NB signs and symptoms vary greatly as some cases can be asymptomatic, while some cases may have non-specific clinical signs such as fever, fatigue, and weight loss [2]. In addition, if the tumor affects the superior cervical ganglia, the patient may develop Horner syndrome [2]. The International Neuroblastoma Staging System (INSS) is based on surgical staging and is utilized in the United States. It is categorized into four stages based on the localization and the metastases of the tumor [2].

Neuroblastoma commonly metastasizes to bone (60%), regional lymph nodes (45%), orbit (20%), liver (15%), brain (14%), and lungs (10%) [4]. Metastasis to the orbits has been well documented for NBs presenting with various symptoms such as periorbital ecchymosis (raccoon eyes) and proptosis. However, NB cases with initial symptoms of orbital involvement are very rare, accounting for approximately 8% of all NB cases [5]. This is a case of a 16-month-old girl with stage IV NB originating from the adrenal gland who presented with proptosis and orbital invasion as the initial symptoms.

## Case presentation

**Figure 1:**
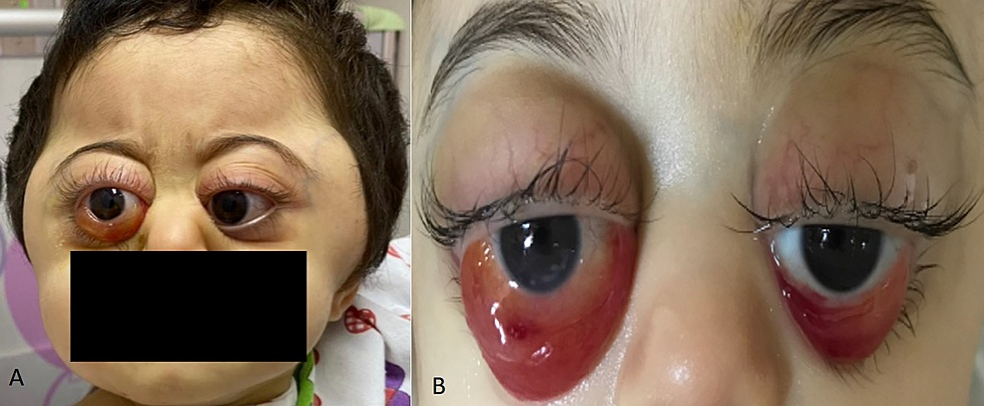
Face photograph showing bilateral proptosis, chemosis, and conjunctival prolapse (A) at initial presentation and (B) rapid progression one week later.

A 16-month-old girl presented to the emergency room with a history of sudden bilateral progressive proptosis for a duration of 15 days. It was associated with decreased activity, reduced oral intake, and weight loss that were noticed by her mother. She was previously healthy with an unremarkable past medical history. There was no history of recent illness, allergies, or trauma. A family history of similar medical conditions was negative. Examination of the eyes showed bilateral severe non-axial, non-pulsatile proptosis that is more prominent in the right eye and was associated with conjunctival prolapse, chemosis, and subconjunctival hemorrhage (Figure 1).

There was a limitation in the extraocular movement, especially on abduction in both eyes but was able to fixate on the target normally. Furthermore, intraocular pressure was 20 mmHg in both eyes and pupil exam showed bilateral reactive pupils with right trace relative afferent pupillary defect. Corneal examination showed evidence of exposure keratopathy in both eyes and the dilated fundus examination showed papilledema. The right optic disc showed grade 3 edema while the left optic disc showed grade 2 edema. Retinal blood vessels tortuosity was seen in both eyes with no signs of retinal infiltration, tumor, or detachment.

Systemic examination, which was performed by a pediatrician, showed bilateral non-tender hard palpable masses on both sides of the head over the temporal bone. Ear-nose-throat examinations were clear. Chest auscultation showed bilateral symmetrical clear air entry. On central nervous system evaluation, she was conscious alert, with no sign of meningeal irritation. Cardiovascular system evaluation was unremarkable. Abdominal examination showed a palpable firm large abdominal mass on the right side crossing the midline, extending 6 cm below the costal margin. The laboratory investigation included routine blood and urine tests, hepatic and renal function tests, and serum glucose tests. All investigations were unremarkable except for normocytic normochromic anemia with a hemoglobin level of 7.9 g/dL.

**Figure 2:**
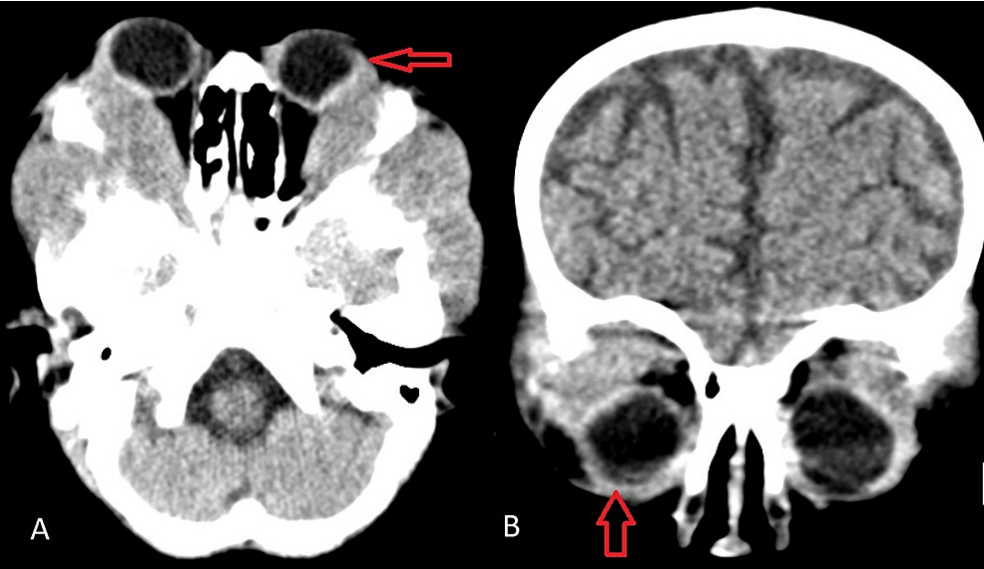
Brain and orbits CT -- (A) axial view with signs of orbital hyperdensity. (B) Coronal view showing bilateral heterogeneous hyperdense lesion in the lateral part of the orbits.

The CT scan of the brain and orbits showed bilateral, non-axial symmetrical heterogeneous hyper-dense lesions at the lateral part of the orbits with bilateral intraconal extension. In the right orbit, the mass extended posteriorly with signs of optic nerve compression at the orbital apex. Laterally, the mass extended into the temporalis muscle (Figure 2).

**Figure 3:**
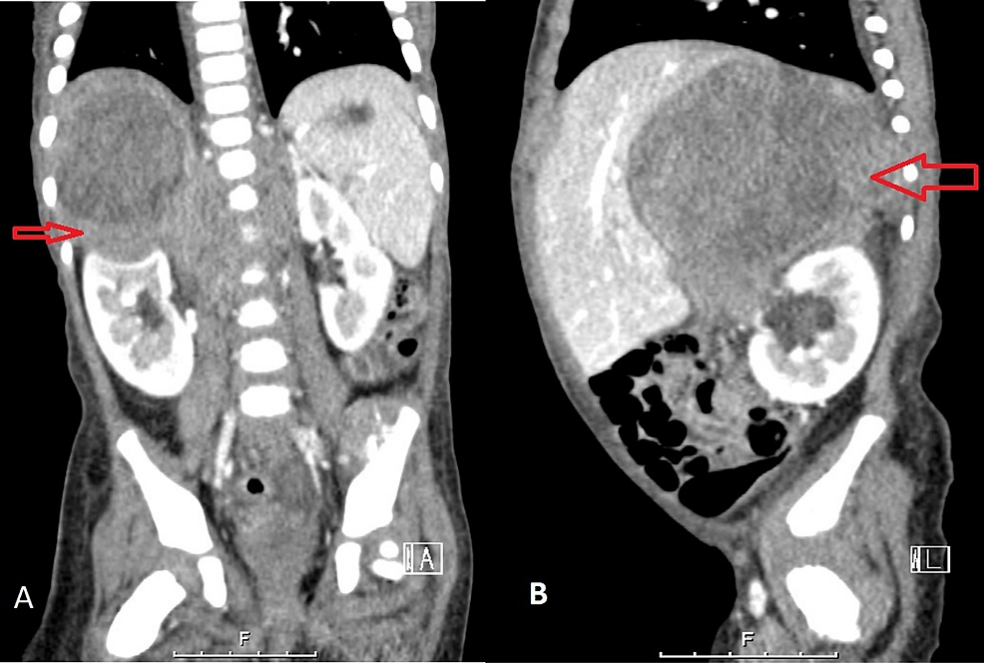
Abdominal and pelvic CT with contrast (A) coronal and (B) sagittal, showing large heterogeneously enhancing retroperitoneal mass arising from the right adrenal gland representing neuroblastoma.

Contrast-enhanced CT of the abdomen and pelvis showed a large heterogeneously enhancing retroperitoneal mass measuring 5.2 cm x 9.0 cm x 10.2 cm in its maximum antero-posterior, transverse and craniocaudal dimension, respectively. It is predominantly located at the right suprarenal region, crossing the midline. It was causing a significant mass effect on the adjacent structures causing anterior displacement of the liver, pancreas, and descending aorta as well as inferior displacement of bowel loops and the right kidney. Findings were compatible with NB arising from the right adrenal gland (Figure 3).

Afterward, an ultrasound-guided abdominal mass biopsy was performed and histopathology evaluation showed a small round blue cell tumor composed of undifferentiated cells with indistinct nucleoli and scant cytoplasm with neuroendocrine features. The immunohاistochemistry showed that the neoplastic cells are positive for CD56, synaptophysin, and chromogranin, and negative to other immunostains. All these findings were in favor of NB. Based on the clinical, radiological, and histopathological findings, the patient was diagnosed with metastatic grade IV NB with orbital invasion. The patient received IV high doses of dexamethasone to relieve compressive optic neuropathy and was closely monitored by the ophthalmology team, and was referred to the pediatric oncology department where she started on chemotherapy protocol for NB (cyclophosphamide/topotecan) as well as supportive treatment.

## Discussion

Ophthalmic involvement occurs in due course of NB in 50% of cases [6]. However, the initial presentation of this tumor with ophthalmic manifestation is rare and few cases were described in the literature as case reports [7-8]. Its ocular manifestations include orbital mass causing proptosis, ecchymosis, Horner’s syndrome, papilledema, nystagmus, extraocular muscle palsy, ptosis, and retinal striae [2-3, 5]. Ophthalmologists' role in recognizing these unique presentations for early diagnosis and proper management is essential as such cases rapidly progress with serious complications such as blindness [7].

 Neuroblastoma represents the second most common orbital tumor in children after rhabdomyosarcoma (RMS). In the present case, the differential diagnosis included NB, RMS, lymphoma, and retinoblastoma (RB). RB was excluded as the fundus examination showed a flat normal retina. Meanwhile, orbital lymphoma is usually present with insidious slowly progressive proptosis which is mostly unilateral with a rare incidence in children as the typical age of presentation is between 50 and 70 years [9]. Therefore, lymphoma was excluded with favor towards NB or RMS due to the rapid progression of the proptosis. On one hand, the usual presentation of RMS is unilateral affecting the older pediatric age group with an average age range between seven and eight years. On the other hand, cases of rapidly progressive proptosis due to NB metastasis have been reported in the literature in children younger than two years of age. Immunohistochemistry plays an important role in the differentiation of different types of tumors and metastatic lesion origin [10]. These include bone metastasis, isolated tumor cells, orbital metastasis, and neuroendocrine tumors [10-11]. In this case, the diagnosis of NB was confirmed with needle biopsy and histopathology.

Treatment of the primary disease is based on the Children’s Oncology Group Neuroblastoma Risk Group Assignment Schema of low, intermediate, or high [12]. As orbital involvement belongs to the high-risk category, aggressive high doses of multi-agent chemotherapy remain the primary approach and is utilized accordingly [12]. Belgaumi et al. suggested high dose multi-agent chemotherapy in infants and children under the age of two for prevention of blindness as such groups have the best chance for resolution [13]. Vallinayagam et al. also reported a case of a rapidly progressive primary orbital NB in a two-year-old patient with an excellent response after completion of three cycles of chemotherapy [8]. Lastly, the prognosis of NB in children is dependent on many factors such as age at diagnosis, disease stage, and histological grade with the highest survival rate found in children under the age of two. However, orbital NB metastases are commonly associated with an overall poor prognosis [14].

## Conclusions

Proptosis in children can be the only manifestation of metastatic orbital NB, especially with rapid progression. Early investigation and systemic work-up should be made immediately, as the metastatic disease is potentially life-threatening that requires aggressive management. The role of the ophthalmologist in these patients depends on the severity of the condition and varies from supportive treatments to active intervention and management of ophthalmic complications in addition to the vital rule in diagnosis and staging as well as assessing response to treatment and long-term follow up.
